# Selenium Nanoparticles Synergistically Stabilized by Starch Microgel and EGCG: Synthesis, Characterization, and Bioactivity

**DOI:** 10.3390/foods12010013

**Published:** 2022-12-20

**Authors:** Jiaojiao Zhou, Yuantao Liu, Yili Hu, Die Zhang, Wei Xu, Lei Chen, Jiangling He, Shuiyuan Cheng, Jie Cai

**Affiliations:** 1 National R&D Center for Se-Rich Agricultural Products Processing, Hubei Engineering Research Center for Deep Processing of Green Se-Rich Agricultural Products, School of Modern Industry for Selenium Science and Engineering, Wuhan Polytechnic University, Wuhan 430023, China; 2 Key Laboratory for Deep Processing of Major Grain and Oil, Ministry of Education, Hubei Key Laboratory for Processing and Transformation of Agricultural Products, Wuhan Polytechnic University, Wuhan 430023, China

**Keywords:** SeNPs, starch microgel, EGCG, apoptosis, anticancer

## Abstract

Selenium (Se) is a chemical element essential to human health because of its bioactive properties, including antioxidative, anticancer, and immunomodulating activities. Despite the high therapeutic potential of Se, its intrinsic properties of poor stability, a narrow therapeutic window, and low bioavailability and bioactivity have limited its clinical applications. Selenium nanoparticles (SeNPs) exhibit lower toxicity and higher bioactivity than other Se forms. Herein, we report a green method for the preparation of monodisperse SeNPs with starch microgel (SM) and epigallocatechin gallate (EGCG) through Se-O bonds and polysaccharide–polyphenol interactions (namely, SM-EGCG-SeNPs). SM-EGCG-SeNPs showed higher stability, bioactivities, and cytotoxicity than SeNPs and SM-SeNPs at the equivalent dose. SM-EGCG-SeNPs induced the apoptosis of cancer cells via the activation of several caspases and reactive oxygen species overproduction. This work proposes a facile method for the design and potentiation of structure-bioactive SeNPs via polysaccharide–polyphenol interactions.

## 1. Introduction

Selenium (Se), a chemical element essential to human health, displays potent bioactivities in cancer prevention and therapy [1,2]. Se could suppress the growth of cancer cells, such as gastric adenocarcinoma, human breast cancer, and nasopharyngeal carcinoma [3,4,5]. However, the extremely narrow boundary between acceptable and toxic doses of Se significantly limits its practical application. It has been confirmed that SeNPs present higher bioavailability than other Se forms [6]. However, SeNPs are unstable and prone to aggregation owing to their high surface energy. Thus, green methods have been proposed for the preparation of SeNPs. Saccharides [7,8], folic acid [9], proteins [10], amino acids [11], and polyphenols [12] have been used to prepare stable SeNPs. These compounds are rich in hydroxyl, amino, carboxyl, and hydrophobic groups, which reduce the surface energy of SeNPs through intermolecular non-covalent bonding [13].

Starch microgel (SM) has become one of the most promising delivery agents owing to its excellent biocompatibility and biodegradability [14,15,16,17]. However, SM is prone to forming aggregates during storage because of its high molecular weight and intermolecular interaction. The introduction of water-soluble polyphenols will be beneficial to the dispersion of SM. Epigallocatechin gallate (EGCG) is the main component of tea polyphenol, and it provides strong antioxidant activity [18,19]. Numerous studies have demonstrated its role in protecting against cancer and other chronic conditions [20,21,22]. On the basis of this, we speculated that incorporating both SM and EGCG into SeNPs may lead to synergistic anti-cancer effects [2,13,23]. Indeed, studies have shown that EGCG can act as a stabilizer and can significantly increase the stability and bioactivity of nanoparticles, when used in combination [12,24,25]. Thus, stabilizing SeNPs with SM and EGCG may be an effective approach to improve their bioactivities.

**Scheme 1:**
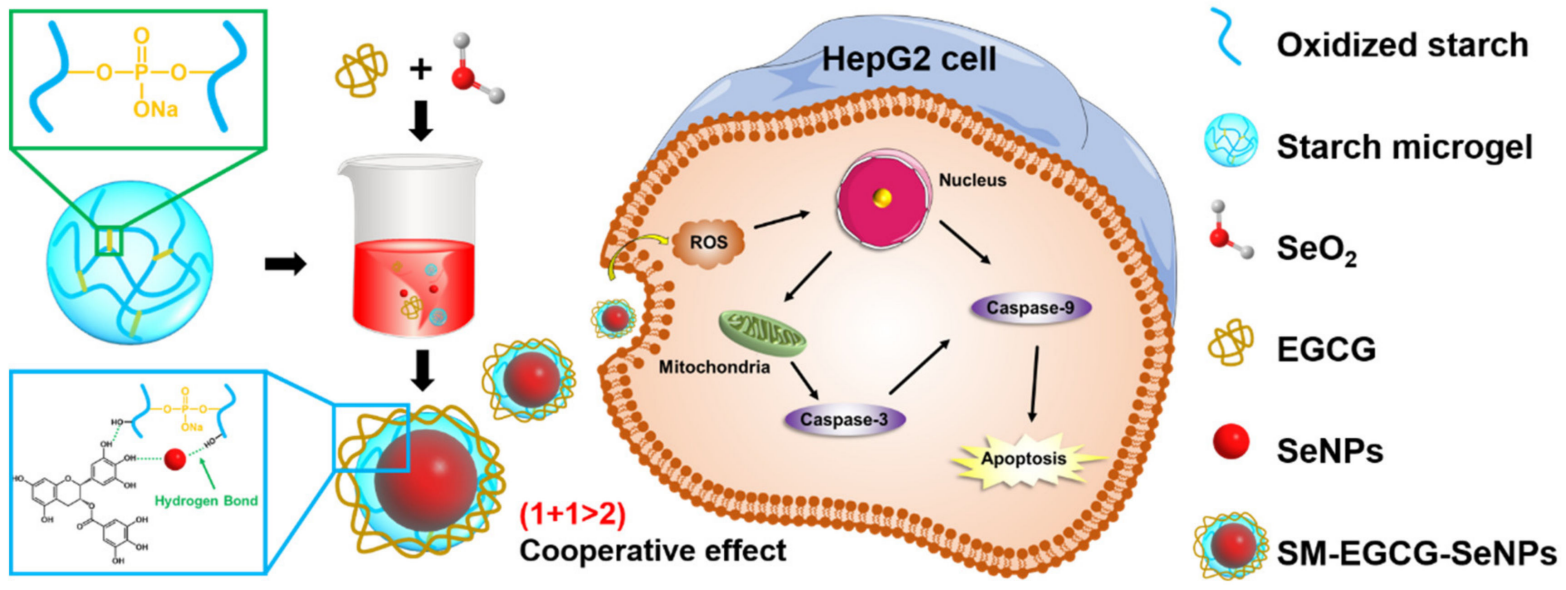
Preparation and working principle of SM-EGCG-SeNPs. SM-EGCG-SeNPs induced apoptosis of cancer cells by activation of several caspases and reactive oxygen species overproduction.

Herein, we report a green method to prepare a Se supplement with improved stability and bioactivity. Both SM and EGCG stabilized SeNPs via Se-O bonding, thus synergistically improving the stability of SeNPs (Scheme 1). In addition, water-soluble EGCG inhibited the self-aggregation of SM. Then, EGCG synergistically enhanced the anticancer activity of SM-SeNPs owing to its stability and bioavailability. SM-EGCG-SeNPs induced apoptosis of cancer cells by the activation of several caspases and reactive oxygen species (ROS) overproduction. Therefore, this study provides a green method to disperse and stabilize SeNPs for the enhancement of the bioactivity and bioavailability of elemental Se. Previously, we have reported the anti-tumor activities of glucan nanoparticles and rosmarinic-acid–stabilized SeNPs [26]. As a continuation, this study focused on the effects of microgel and EGCG on the stability and bioactivity of SeNPs in vitro. This work contributes to the development of Se supplements for food and biomedical applications.

## 2. Materials and Methods

### 2.1. Materials and Instruments

Chemicals and instruments are available in the Supplementary Materials.

### 2.2. Preparation of SM

Oxidized corn starch solution (oxidation degree: 30%) was added with 0.5 g of NaOH and 1.6 g of sodium triphosphate, and the mixture was stirred at 500 rpm, leading to gel formation. The gel was stored at 4 °C for 12 h and passed through a sieve covered with a 200 mesh nylon cloth. The entire gel was washed with deionized water to neutralize and kept overnight at −20 °C. Finally, the microgel products were collected by lyophilization.

### 2.3. Preparation of SM-EGCG-SeNPs

SeNPs were prepared by the reported chemical reduction approach [26]. SM-SeNPs were synthesized by adding ascorbic acid (75 mM) into the above SM solution under stirring. A suspension of 10 mL of 25 mM H_2_SeO_3_ was added to the reaction system. Finally, excessive reactants were removed.

SM-EGCG-SeNPs were obtained by adding EGCG into 100 mL of SM-SeNPs under stirring. After freeze-drying, SM-EGCG-SeNPs were collected.

### 2.4. DPPH Radical Scavenging Assay

The DPPH radical scavenging ability of nanoparticles was determined as reported [27]. Briefly, different concentrations of nanoparticles were mixed with DPPH for 0.5 h for the absorbance record. The DPPH scavenging ability was calculated as

(1) $$\mathrm{DPPH}\;\mathrm{scavenging}\;\mathrm{rate}\;\left(\%\right)=\left(A_{c}-A_{s}+A_{b}\right)/A_{c}\times 100\%$$

where *A_c_*, *A_s_*, and *A_b_* represent the absorbance of the control, the sample mixed with DPPH, and the blank (sample without DPPH), respectively.

### 2.5. Hydroxyl Radical Scavenging Assay

Hydroxyl radical scavenging ability was evaluated according to our previous report [26]. Briefly, various concentrations of nanoparticles were separately added with 9 mM FeSO_4_, 9 mM salicylic acid, and 8.8 mM H_2_O_2_. After 0.5 h incubation, the absorbance of the solution was recorded with a spectrophotometer. The hydroxyl radical scavenging activity was calculated as

(2) $$\mathrm{Hydroxyl}\;\mathrm{scavenging}\;\mathrm{rate}\;\left(\%\right)=\left[1-\left(A_{1}-A_{2}\right)/A_{0}\right]\times 100\%$$

where *A*_0_, *A*_1_, and *A*_2_ represent the absorbance of the control, the test sample, and the blank, respectively.

### 2.6. Cell Viability Assay

Cell viability was assessed according to our previous report [26]. The antiproliferative activity of nanoparticles was evaluated using Cell Counting Kit-8 (CCK-8) assay. Various concentrations of nanoparticles (EGCG, SM, SM-SeNPs, and SM-EGCG-SeNPs) were added into the cultured cells separately and then incubated for 24 h, followed by the addition of CCK-8 solution. After 4 h incubation, the absorbance of the solution was recorded at 405 nm. The percentage of cell viability was calculated as

(3) $$\mathrm{Cell}\;\mathrm{viability}\;\left(\%\right)=\left(A_{s}-A_{c}\right)/A_{b}\times 100\%$$

where *A_s_*, *A_c_*, and *A_b_* represent the absorbance of the sample, control, and blank, respectively.

### 2.7. Analysis of Cell Apoptosis

Cell apoptosis was evaluated using an apoptosis kit according to the manufacturer’s guidelines. The cultured cells were mixed with the apoptosis agents for 15 min incubation after treatment with SM-EGCG-SeNPs, after which flow cytometry analysis was performed.

### 2.8. Activities of Caspase-3 and -9

Activities of caspase-3 and -9 were measured as previously reported [26]. The cultured cells were incubated on ice for 15 min after treatment with SM-EGCG-SeNPs. Afterward, the mixture was centrifuged (15,000 rpm, 10 min), and the supernatants were collected and added with the specific caspase substrates. Subsequently, the caspase activity was measured with the absorbance at 405 nm.

### 2.9. ROS Production

ROS production was recorded using a 2,7-dichlorofluorescin diacetate (DCFH-DA) probe as previously reported [28]. In brief, the treated cells were mixed with DCFH-DA for 2 h, and the resulting solution was monitored with a fluorescence spectrometer (Ex: 488 nm; Em: 525 nm).

## 3. Results

### 3.1. Characterization of SM-EGCG-SeNPs

**Figure 1:**
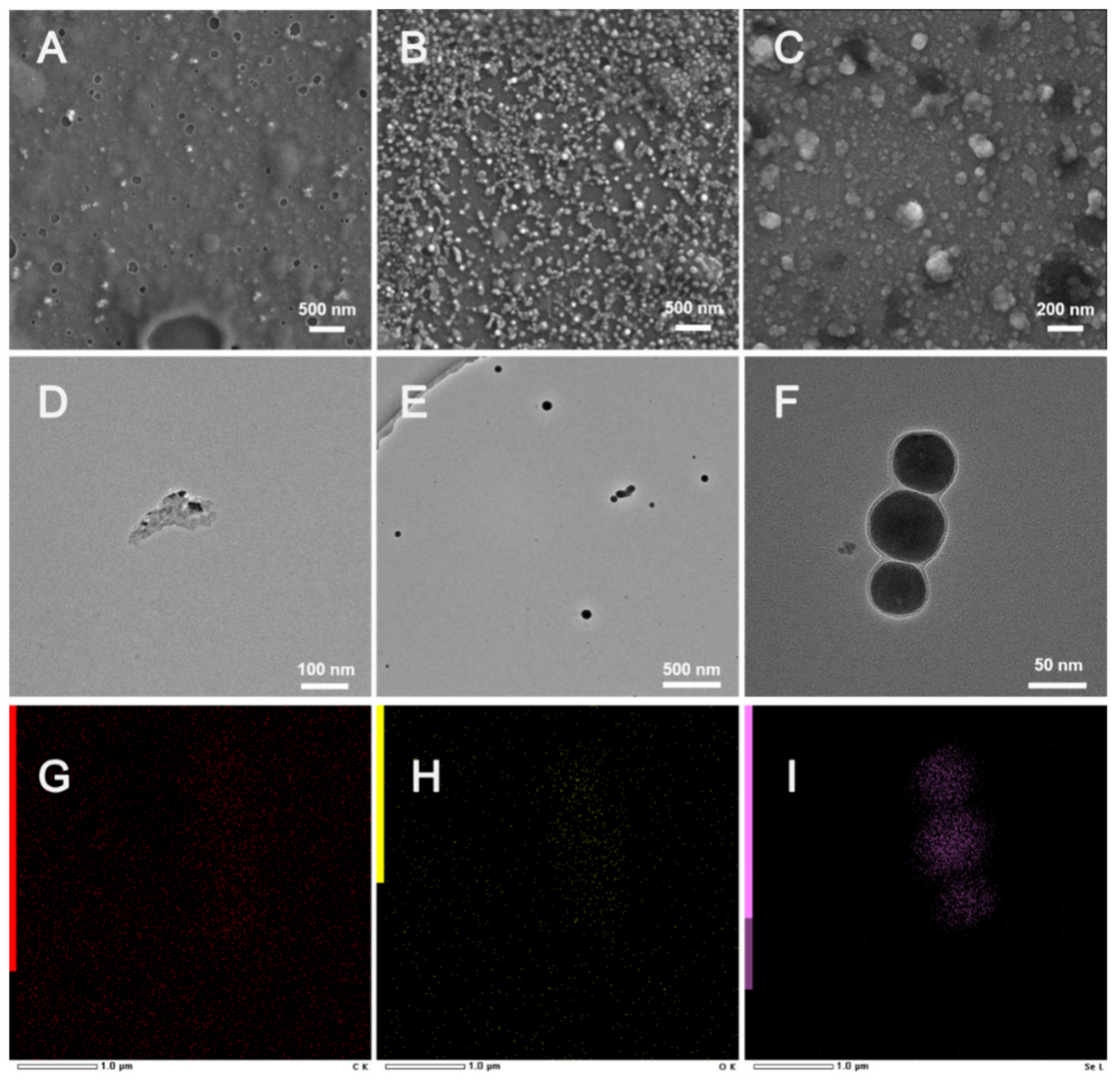
SEM images of (**A**) SM, (**B**) SM-SeNPs, and (**C**) SM-EGCG-SeNPs. TEM images of (**D**) SM, (**E**) SM-SeNPs, and (**F**) SM-EGCG-SeNPs. EDS elemental mapping of (**G**) C, (**H**) O, and (**I**) Se.

The morphology of SM-EGCG-SeNPs was evaluated using TEM and SEM. As shown in Figure 1A,D, SM was spherical with a diameter of approximately 200 nm, whereas SeNPs synthesized without a template were coral-like because of their poor solubility and high specific surface area (Figure S1, Supplementary Materials). After adding SM as a template, SM-SeNPs had a uniform diameter of 82.1 ± 3.3 nm (Figure 1B,E), and a core–shell nanostructure was observed for SM-EGCG-SeNPs in the presence of SM and EGCG (Figure 1C,F), indicating the importance of SM and EGCG in the dispersion and stability of SeNPs. Moreover, energy-dispersive spectroscopy (EDS) elemental mapping analysis (Figure 1G–I) showed the C, O, and Se elemental distribution in SM-EGCG-SeNPs. The results demonstrate the successful synthesis of SM-EGCG-SeNPs.

The chemical structure of SM-EGCG-SeNPs was investigated with UV-vis, FT-IR, XPS, and XRD (Figure S2, Supplementary Materials). The UV-vis spectra showed peaks at 260 nm, 254 nm, and 272 nm for ascorbic acid, SeNPs, and EGCG, respectively. Notably, a peak appeared at 274 nm for SM-SeNPs after introducing EGCG, which was comparable to the characteristic absorption peak of EGCG. The absorption intensity of SM-EGCG was weaker than that of EGCG, owing to the complexation of polyphenols with polysaccharide [29]. This micromolecule–macromolecule interaction between polyphenols and polysaccharides plays a key role in inhibiting SM aggregation. The peaks between 3300 and 3500 cm^−1^ were attributed to the O-H stretching vibration. An obvious shift from 3405 cm^−1^ to 3415 cm^−1^ and 3417 cm^−1^ appeared in SM-SeNPs and SM-EGCG-SeNPs, respectively, indicating that SeNPs mainly reacted with the hydroxyl groups of SM. The absorption peak of SM at 1024 cm^−1^ was related to O-H stretching vibration, which shifted to 1033 cm^−1^ (SM-SeNP peak) and 1031 cm^−1^ (SM-EGCG-SeNP peak), and the peak intensity significantly decreased, indicating an interaction between the hydroxyl groups of SM and SeNPs via Se-O bonding. The peak at 1455 cm^−1^ was correlated with C-H bending vibration. In particular, the peaks at 1536 cm^−1^, 1455 cm^−1^, and 1235 cm^−1^ confirmed the existence of ECCG in SM-EGCG-SeNPs. The results indicate that both SM and EGCG stabilize SeNPs via intermolecular Se-O bonds [30]. In addition, the XPS spectra revealed that the 3d orbital binding energy of SeNPs, SM-SeNPs, and SM-EGCG-SeNPs was about 55.08 eV. These results indicate the zero-valent state of SeNPs in the SM-EGCG-SeNP nanosystem. Furthermore, the binding energy of Se 3d_5/2_ decreased in both SM-SeNPs and SM-EGCG-SeNPs compared to that of SeNPs. An increase in the binding energy of Se 3d_3/2_ in SM-SeNPs was observed, compared to SeNPs, whereas a decrease in SM-EGCG-SeNPs was observed when EGCG was further introduced. This suggests the involvement of Se 3d in Se binding with SM and EGCG. Notably, in the fitting spectrum of O 1s, the peak in both SM-SeNPs and SM-EGCG-SeNPs split into two, whereas there was only one peak in the fitting spectra of both SM and EGCG. A shift from 532.54 eV (SM peak) and 533.03 eV (EGCG peak) to 533.43 eV (SM-EGCG-SeNP peak) was observed, indicating the formation of Se-O bonds. These results illustrate the successful preparation of SM-EGCG-SeNPs. The XRD spectra of Se powder exhibited strong diffraction peaks at 24°, 30°, 41.2°, 43.5°, and 51.6°, indicating its crystalline structure. However, the XRD pattern of SM-EGCG-SeNPs was noisy and broad without sharp reflections, which is indicative of the noncrystalline nature of the SeNPs.

**Figure 2:**
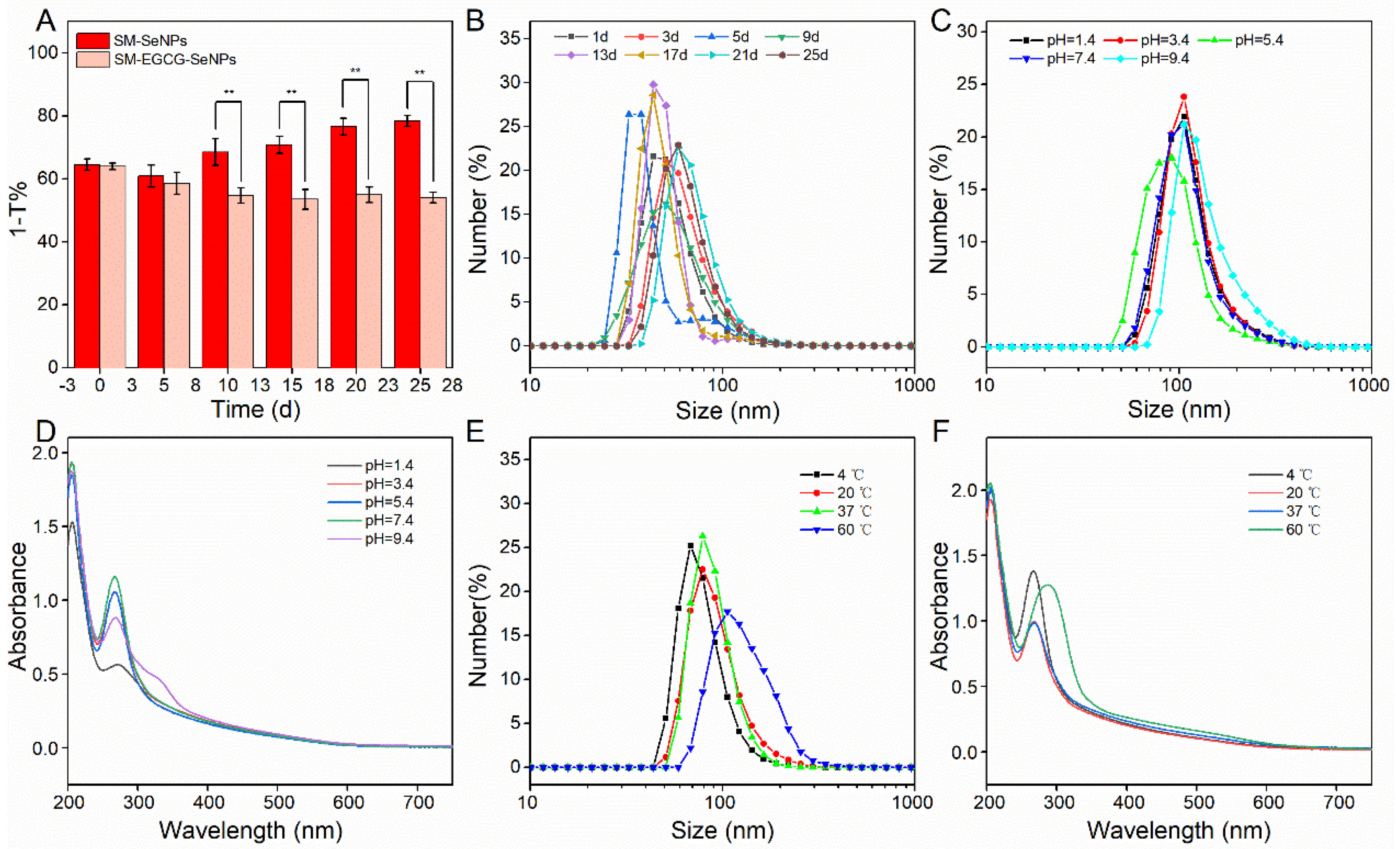
Characterization of SeNPs system. (**A**) Turbidity changes of SM-SeNPs and SM-EGCG-SeNPs. (**B**) Particle size variation in SM-EGCG-SeNPs during storage. Changes in (**C**) particle size and (**D**) UV-vis absorption spectra of SM-EGCG-SeNPs versus pH values. Changes in (**E**) particle size and (**F**) UV-vis absorption spectra of SM-EGCG-SeNPs versus temperature. The difference between groups is indicated at ** *p* < 0.01.

The colloidal stability in biological solutions is an important factor for nanoparticles. The freshly prepared SeNP solution was red and cloudy, while it became transparent red when introducing SM and EGCG (Figure S3, Supplementary Materials). Obvious aggregates of SeNPs were observed after the addition of templates. The solution of SM-SeNPs became cloudy with prolonged storage, but the color remained red. Interestingly, the solution of SM-EGCG-SeNPs became more transparent after 25 days. These results indicate that these nanoparticles were well-dispersed even after 25 days. We proceeded to investigate the change in turbidity of SM-SeNPs and SM-EGCG-SeNPs over time. The turbidity of SM-SeNPs increased over time, which might have resulted from the collision-mediated aggregation of SM-SeNPs [31]. However, the turbidity of SM-EGCG-SeNPs decreased over time (Figure 2A), which might be attributed to the inhibition of SM aggregation by EGCG. With the continuous interaction among SeNPs, EGCG, and SM in the system, the turbidity remained stable after 25 days, which fully reflects the synergistic dispersion effect of EGCG. The stability tests revealed the increased size of SM-SeNPs from 85.81 ± 1.1 nm to 151.63 ± 0.76 nm (Figure S4, Supplementary Materials), whereas the size distribution of SM-EGCG-SeNPs changed slightly (from 83.59 ± 2.81 nm to 98.74 ± 0.98 nm) during the 25-day storage period (Figure 2B). This indicates the high stability of SM-EGCG-SeNPs. It is worth noting that the size variation in SM-SeNPs was consistent with the turbidity of SM-SeNPs. In comparison, the turbidity of SM-EGCG-SeNPs decreased, whereas its particle size increased by 12.13 nm. This finding further confirmed that EGCG might inhibit the aggregation of SM via non-covalent interaction [32,33]. The stability of the SM-EGCG-SeNP system under different pH conditions was also investigated. The size of SeNPs slightly varied within the pH range of 5.4–9.4, and it sharply increased to 220 ± 6.35 nm at pH 3.4 (Figure S5A, Supplementary Materials). Noticeably, the diameter was undetectable at pH 1.4, suggesting the instability of SeNPs at low pH. In contrast, SM-SeNPs had diameters of 142.6 ± 3.42 and 124.3 ± 1.94 nm at pH 1.4 and 3.4, respectively (Figure S5B, Supplementary Materials). In contrast, SM-EGCG-SeNPs were relatively stable within a pH range from 1.4 to 9.4 (Figure 2C). This suggests that SM-EGCG-SeNPs have significantly higher stability than SM-SeNPs. These findings imply that the introduction of EGCG further stabilizes SM-SeNPs at low pH [34]. SM-SeNPs and SM-EGCG-SeNPs presented similar UV-vis absorption profiles in the pH range of 1.4–9.4 (Figure 2D and Figure S6, Supplementary Materials). Changes in the particle size and UV–vis absorption spectra of SM-SeNPs (Figure S7, Supplementary Materials) and SM-EGCG-SeNPs (Figure 2E,F) were examined within a temperature range of 4–60 °C. The average diameter and UV–vis absorption spectra of SM-EGCG-SeNPs were observed to vary slightly with temperature, indicating their high stability. These results demonstrate that both SM and EGCG can enhance the stability of SeNPs.

### 3.2. Evaluation of Antioxidant Activity of SM-EGCG-SeNPs

Antioxidant ability is an important factor when evaluating the value of Se supplements and is routinely characterized by the free radical scavenging ability (Figure S8, Supplementary Materials). SM-SeNPs showed a slight increase in DPPH free radical scavenging activity with an increase in SM-SeNP concentration from 1.44 to 91.75 μg/mL. EGCG presented stronger scavenging activities than that of SM-SeNPs at every concentration point. In contrast, SM-EGCG-SeNPs exhibited a superior scavenging activity of 85.64%, which is better than that of free EGCG molecules.

The hydroxyl free radical scavenging abilities of EGCG, SM-SeNPs, and SM-EGCG-SeNPs also showed similar trends. The hydroxyl radical scavenging activity of SM-SeNPs displayed a concentration-dependent manner. At concentrations from 22.94 to 91.75 μg/mL, the scavenging rate of SM-EGCG-SeNPs dramatically increased and was slightly higher than that of EGCG.

Moreover, according to the above results, the synergetic effect of SeNPs and EGCG during free radical scavenging processes was determined with isobologram analysis. The SM-EGCG-SeNPs appeared below the additive curve, suggesting the existence of synergistic effects between SeNPs and EGCG in the SM-EGCG-SeNPs. This result demonstrates that polyphenols could enhance the bioactivities of SeNPs.

### 3.3. Antiproliferative Effect of SM-EGCG-SeNPs

**Figure 3:**
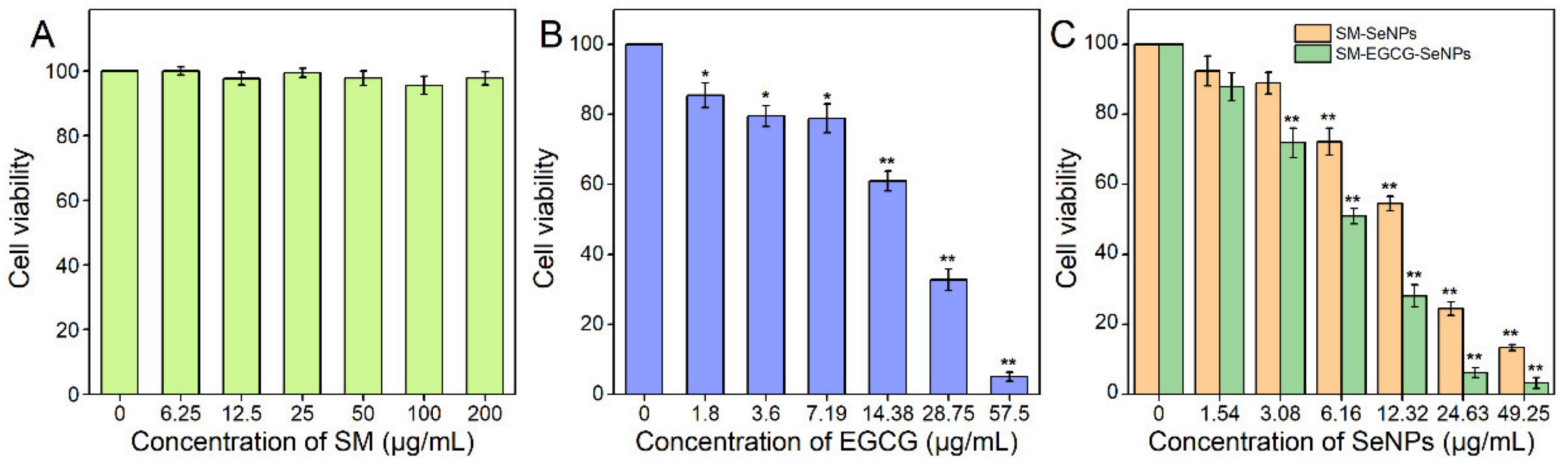
Antiproliferative effect of (**A**) SM, (**B**) EGCG, (**C**) SM-SeNPs and SM-EGCG-SeNPs. Each value represents the mean ± SD. The difference between groups is indicated at * *p* < 0.05 and ** *p* < 0.01.

HepG2 cells were used to evaluate the antiproliferative activities of SM, EGCG, SM-SeNPs, and SM-EGCG-SeNPs. EGCG, SM-SeNPs, and SM-EGCG-SeNPs exhibited dose-dependent inhibition toward HepG2 cells, with IC_50_ (50% cell viability) values of 19.56, 14.19, and 6.31 μg/mL, respectively (Figure 3). By contrast, SM displayed very limited inhibition on HepG2 cells, certifying its superior biocompatibility. Notably, the inhibition rate of SM-SeNPs and SM-EGCG-SeNPs on HepG2 cells increased to 86.67% and 96.76% at 49.25 μg/mL, respectively. Compared to the SM, EGCG, and SM-SeNP treatment groups, the SM-EGCG-SeNP group showed the highest antiproliferative activity toward HepG2 cells. These results suggest that polyphenol modification could improve the antiproliferative activity of SM-SeNPs.

### 3.4. Cell Apoptosis Induction Caused by SM-EGCG-SeNPs in HepG2 Cells

**Figure 4:**
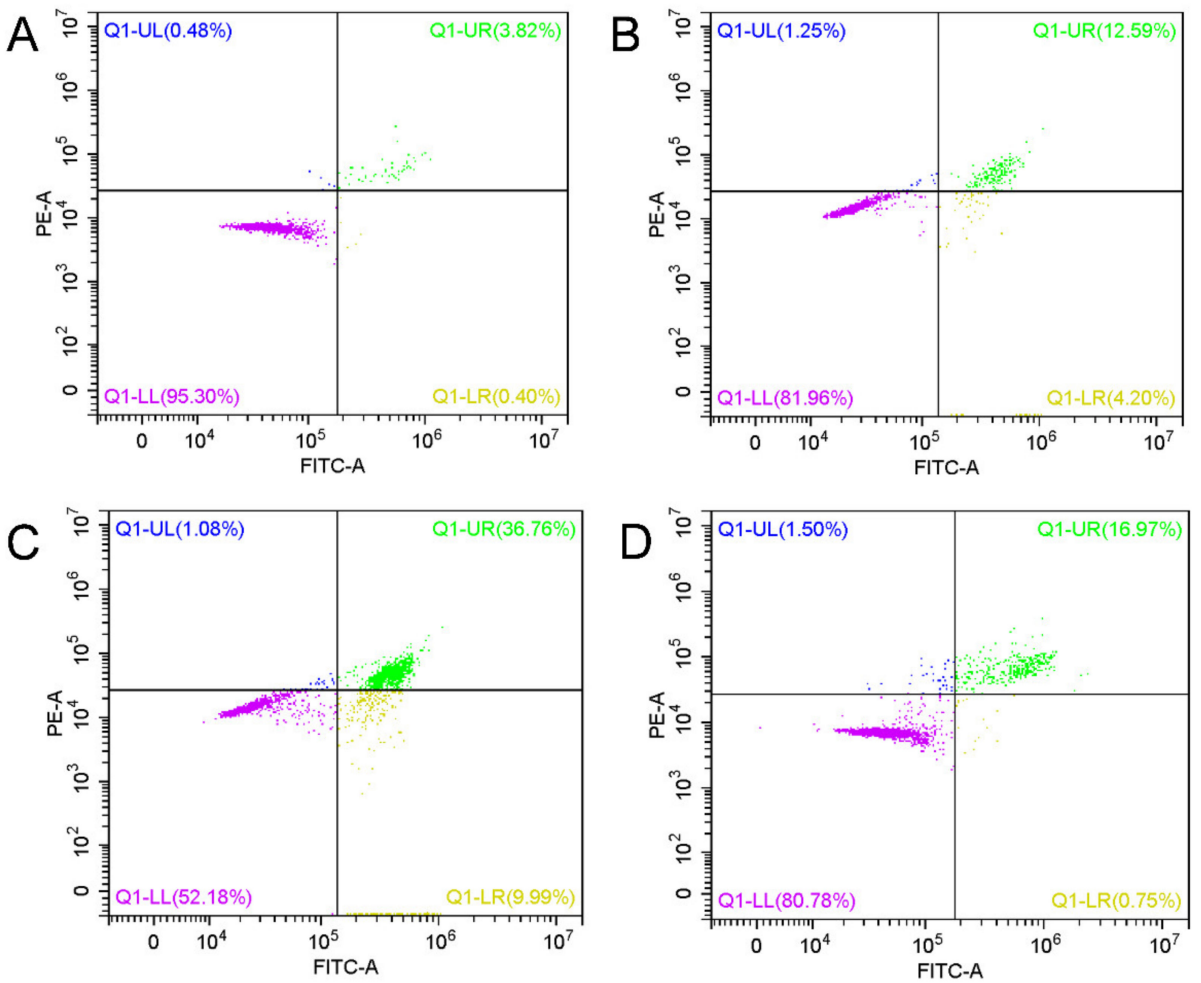
Cell apoptosis after treatment with SeNPs system observed via flow cytometry: (**A**) control, (**B**) SM-SeNPs, (**C**) SM-EGCG-SeNPs, and (**D**) EGCG.

Apoptosis is a genetically controlled process of programmed cell death [35]. Previous studies have reported that Se-based materials exhibited potent antitumor activity via an apoptotic mechanism [26,36]. To elucidate the effects of SM-EGCG-SeNPs on cancer cell apoptosis, after treatment with SM-SeNPs, SM-EGCG-SeNPs, and EGCG, the apoptosis cells increased from 4.22% to 16.79%, 46.75%, and 17.73%, respectively, as compared with those in the control groups (Figure 4). Flow cytometry proved that SeNPs and EGCG could induce cell apoptosis. Collectively, the cytotoxicity data clearly demonstrate improved apoptosis efficacy of SeNPs upon the introduction of EGCG.

**Figure 5:**
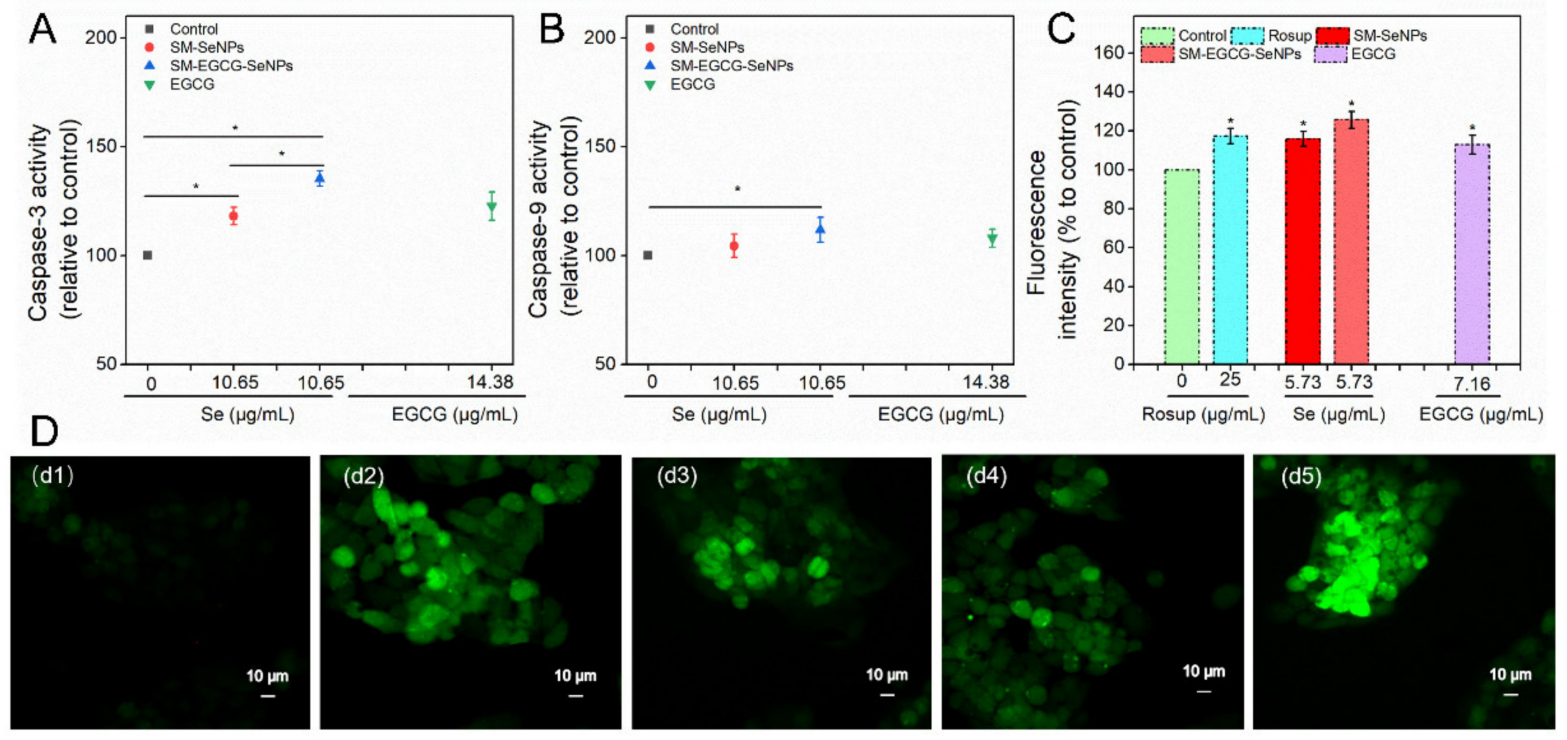
Activities of caspase-3 (**A**) and caspase-9 (**B**) in HepG2 cells in the SM-SeNP group, EGCG group, and SM-EGCG-SeNP group, respectively. (**C**) ROS analysis. (**D**) Confocal laser images of ROS production: (d1) blank, (d2) positive, (d3) SM-SeNPs, (d4) EGCG, and (d5) SM-EGCG-SeNPs. Each value represents the mean ± SD. The difference between groups is indicated at * *p* < 0.05.

Caspases are involved in cell apoptosis, and their activation can lead to cell apoptosis [37]. To prove the possible mechanism of caspase-dependent apoptosis, the activities of caspase-3 and -9 were detected. After treatment with SM-SeNPs or SM-EGCG-SeNPs, the activities of caspase-3 and -9 in HepG2 cells significantly increased (Figure 5A,B). These results demonstrate that SM-EGCG-SeNPs can induce higher levels of cell apoptosis than SM-SeNPs and EGCG via activating caspase-3 and -9. Thus, we report caspase-dependent apoptosis as a potential mechanism of tumor impression caused by Se.

It has been reported that the antitumor activities of Se may be related to ROS-m1ediated cell apoptosis [38,39]. To further elucidate whether ROS is involved in the process of SM-EGCG-SeNP-induced cell apoptosis, ROS formation was determined using a DCFH-DA probe. After 2 h of treatment, the ROS levels in HepG2 cells significantly increased (Figure 5C). More importantly, the ROS level in the SM-EGCG-SeNP group (12.68%) was higher than that in the SM-SeNP group (11.59%) and EGCG treatment (11.30%) due to the SeNP metabolism and EGCG oxidation. Confocal scanning microscopy analysis further confirmed the ROS production in tumor cells (Figure 5D). Therefore, these results not only verify the key role of ROS generation by SM-EGCG-SeNP-induced apoptosis but also illuminate the potential mechanism of caspase-dependent apoptosis.

## 4. Conclusions

In conclusion, structure-stable and bioactive SeNPs were obtained using SM and EGCG as templates (namely, SM-EGCG-SeNPs). Core–shell structural SeNPs bond with SM and EGCG through Se-O bonds. SM-EGCG-SeNPs displayed a synergistic enhancement in the cytotoxicity of HepG2 cells compared to EGCG and SM-SeNPs. Cell experiments indicated that SM-EGCG-SeNPs have potential anticancer efficacy via the induction of apoptosis. SM-EGCG-SeNPs induced the apoptosis of cancer cells via a caspase-dependent apoptosis pathway in conjunction with ROS generation. As a result, these novel nanoparticles have potential applications in the biomedicine field. However, it is important to obtain regulatory approval before this new nano-selenium system is widely used in the food industry.

## Data Availability

The data presented in this study are available on request from the corresponding author. The data are not publicly available due to privacy.

## Supplementary Materials

The following supporting information can be downloaded at: https://www.mdpi.com/article/10.3390/foods12010013/s1, Figure S1: SEM of SeNPs; Figure S2: Characterization of SeNPs system; Figure S3: Photographs of aqueous solution. Figure S4: Variation in particle size; Figure S5: Changes in particle size; Figure S6: Changes in UV-vis absorption spectra; Figure S7: Changes in particle size and UV-vis absorption spectra; Figure S8: DPPH radical scavenging activities.
