# Supplementary material for: Selenium Nanoparticles Synergistically Stabilized by Starch Microgel and EGCG: Synthesis, Characterization, and Bioactivity
Source: Foods. 2022 Dec 20;12(1):13. doi: 10.3390/foods12010013 (PMC9818717; doi:10.3390/foods12010013)
Supplement: Supplementary file 1 [file foods-12-00013-s001.zip › foods-2023760-supplementary.pdf]

Supplementary Materials

Selenium Nanoparticles Synergistically Stabilized by Starch Microgel and EGCG: Synthesis, Characterization and Bioactivity

Jiaojiao Zhou [1], Yuantao Liu [1], Yili Hu [1], Die Zhang [2], Wei Xu [2], Lei Chen [2], Jiangling He [1], Shuiyuan Cheng [1] and Jie Cai [1,2,*]

[1] National R&D Center for Se-Rich Agricultural Products Processing, Hubei Engineering Research Center for Deep Processing of Green Se-Rich Agricultural Products, School of Modern Industry for Selenium Science and Engineering, Wuhan Polytechnic University, Wuhan 430023, China

[2] Key Laboratory for Deep Processing of Major Grain and Oil, Ministry of Education, Hubei Key Laboratory for Processing and Transformation of Agricultural Products, Wuhan Polytechnic University, Wuhan 430023, China

* Correspondence: caijievip@whpu.edu.cn

Experimental Section

1. Materials and Reagents

Salicylic acid, ascorbic acid, $FeSO_4$, and 1,1-diphenyl-2-picrylhydrazyl (DPPH) were from Aladdin Reagent Co., Ltd (Shanghai, China). EGCG and oxidized starch were purchased from Shanghai Yuanye Bio-Technology Co., Ltd. (Shanghai, China). The human hepatocarcinoma cell line (HepG2) was provided by China Center for Type Culture Collection (CCTCC, China). RPMI 1640 medium, trypsin, and antibiotics were purchased from Thermo Fisher Scientific Chemicals, Inc. (USA). Cell counting kit-8 (CCK-8) kit, annexin V-FITC apoptosis detection kit, ROS assay kit, and caspase activity assay kit were obtained from Shanghai Beyotime Bio-Tech Co., Ltd. (Shanghai, China). All the other chemical reagents used in the present study were of analytical grade.

2. Instrumentation

The morphologies of nanoparticles were determined by scanning electron microscopy (SEM) (Gemini300) and transmission electron microscopy (TEM) (JEOLJEM-2100F). The element distribution of SM-EGCG-SeNPs was measured by energy-dispersive X-ray spectroscopy (EDS) element mapping. Hydrodynamic diameters were measured on a Malvern Zetasizer Nanoseries (Malvern, England). Fourier transform infrared (FT-IR) spectra were recorded with a Thermo Nicolet NEXUS670 spectrometer. The crystalline phases were detected by an X-ray diffractometer (Empyrean) using Cu-Kα radiation within the 2θ range of 10° to 60°. Surface element analysis was conducted on an X-ray photoelectron spectroscopy (XPS) instrument (Thermo Scientific K-Alpha).

Supporting Figures

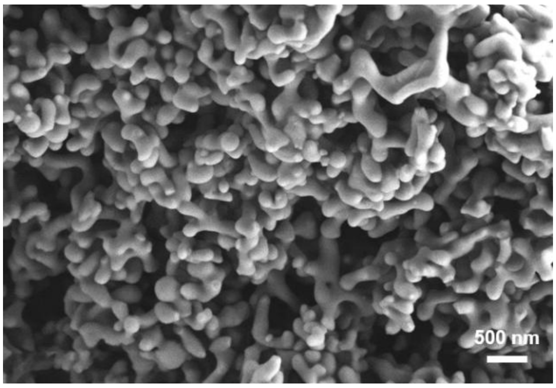

Figure S1. SEM of SeNPs.

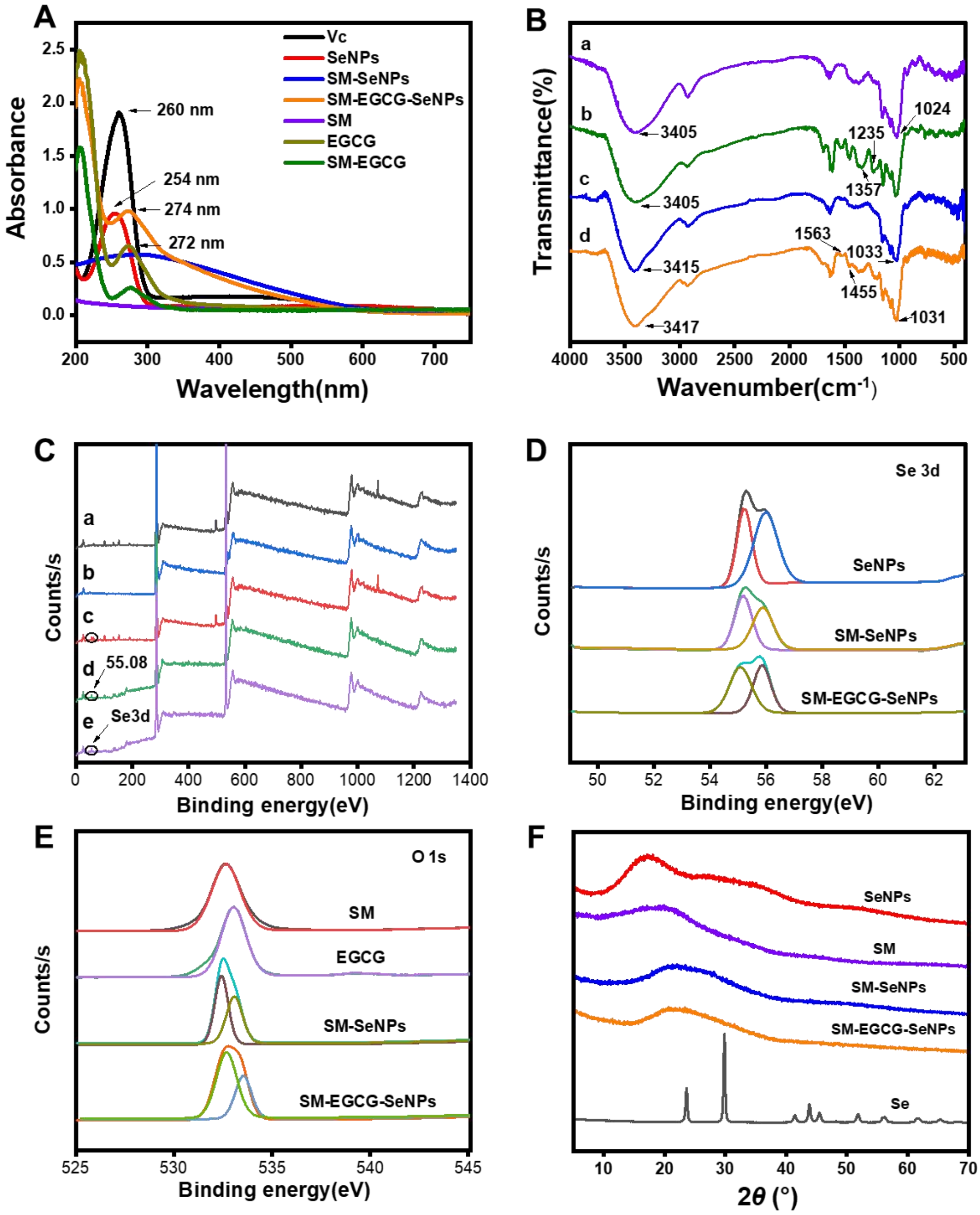

Figure S2. Characterization of SeNPs system. (A) UV-vis absorption spectra, (B) FT-IR spectra (a: SM; b: SM-EGCG; c: SM-SeNPs; d: SM-EGCG-SeNPs), (C, D, E) XPS spectra (a: SM; b: EGCG; c: SeNPs; d: SM-SeNPs; e: SM-EGCG-SeNPs), and (F) XRD curves.

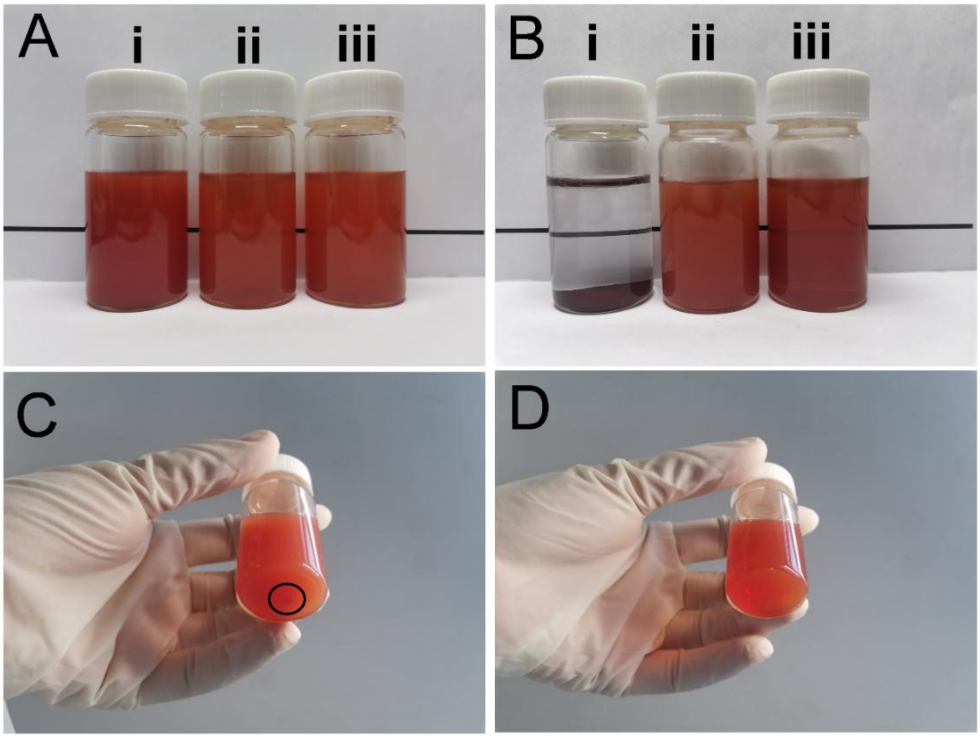

Figure S3. Photographs of aqueous solution of (i) SeNPs, (ii) SM-SeNPs, and (iii) SM-EGCG-SeNPs at 0 d (A) and at 25 d (B). Photographs of SM-SeNPS (C) and SM-EGCG-SeNPS in 25 d (D).

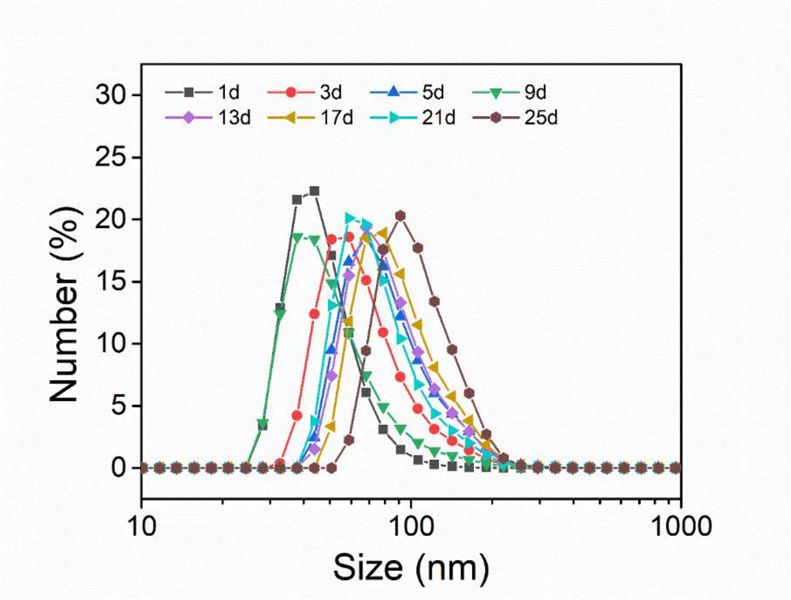

Figure S4. Variation in particle size of SM-SeNPs system with time.

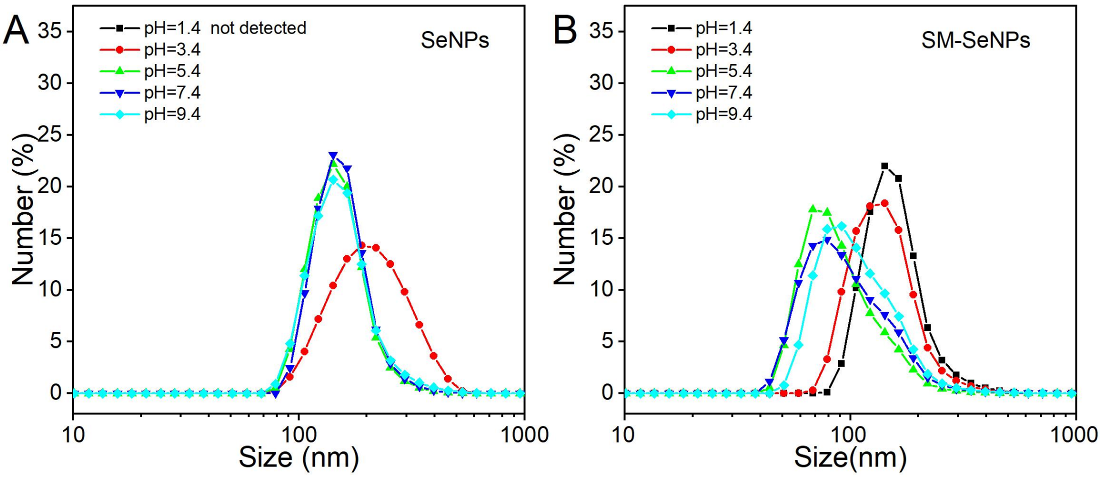

Figure S5. Changes in particle size of SeNPs (A) and SM-SeNPs (B) under different pH conditions.

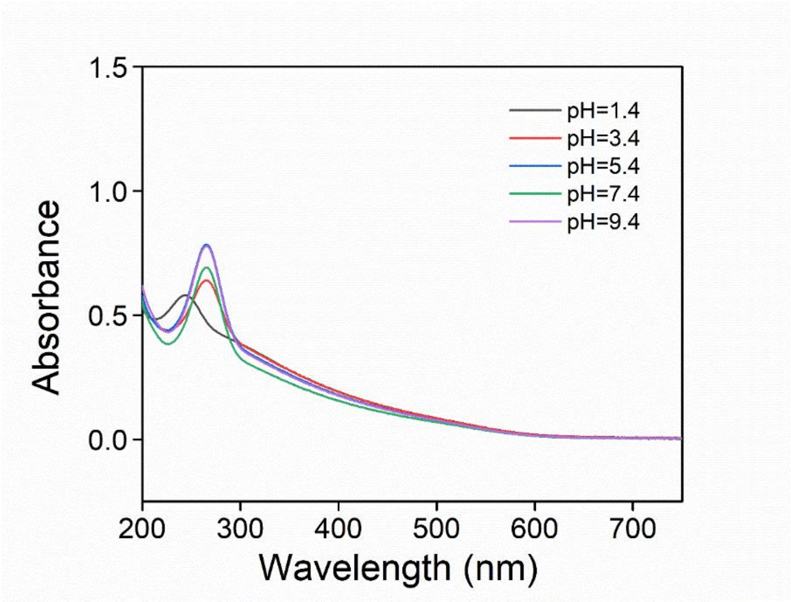

Figure S6. Changes in UV-vis absorption spectra of SM-SeNPs versus pH values.

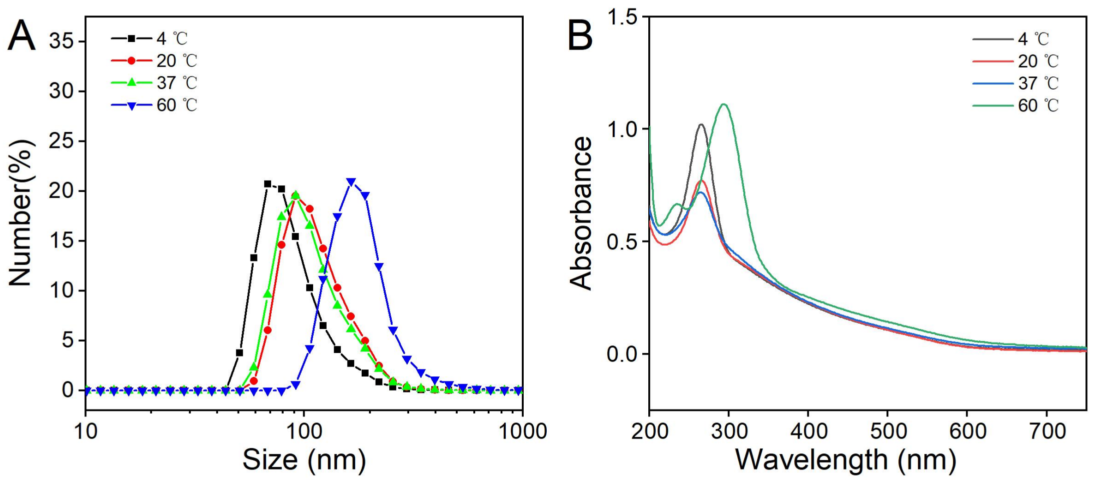

Figure S7. Changes in (A) particle size and (B) UV-vis absorption spectra of SM-SeNPs versus temperature.

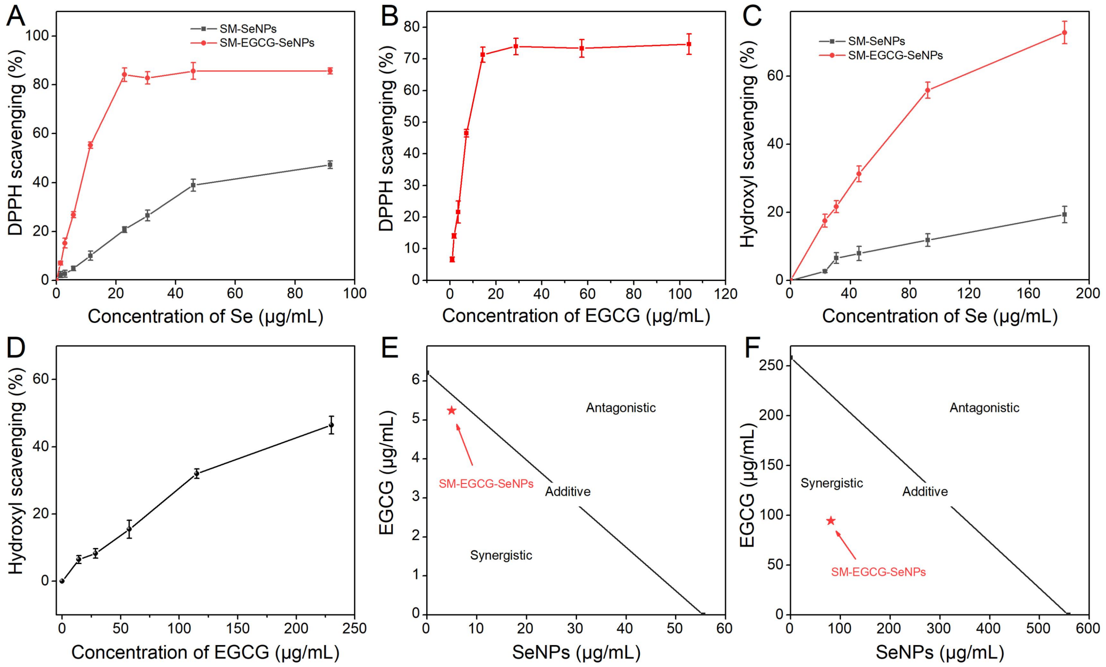

Figure S8. DPPH radical scavenging activities of (A) SM-SeNPs and SM-EGCG-SeNPs, and (B) EGCG. Hydroxyl radical scavenging abilities of (C) SM-SeNPs and SM-EGCG-SeNPs, and (D) EGCG. Isobologram analysis of (E) DPPH radical and (F) hydroxyl radical equivalence scavenging by SeNPs and EGCG, respectively.
